# A Survey on Nutritional Knowledge in Coeliac Disease Compared to Inflammatory Bowel Diseases Patients and Healthy Subjects

**DOI:** 10.3390/nu12041110

**Published:** 2020-04-16

**Authors:** Ilaria Marsilio, Edoardo Vincenzo Savarino, Brigida Barberio, Greta Lorenzon, Daria Maniero, Linda Cingolani, Anna D’Odorico, Renata D’Incà, Fabiana Zingone

**Affiliations:** Division of Gastroenterology, Department of Surgery, Oncology and Gastroenterology, University of Padua, Via Giustiniani 2, 35121 Padua, Italy; ilaria.marsilio@gmail.com (I.M.); brigida.barberio@gmail.com (B.B.); gretalorenzon90@gmail.com (G.L.); dariamaniero@gmail.com (D.M.); linda.cingo@gmail.com (L.C.); anna.dodorico@aopd.veneto.it (A.D.); dinca@unipd.it (R.D.); fabiana.zingone@unipd.it (F.Z.)

**Keywords:** coeliac disease, IBD, nutritional knowledge, diet

## Abstract

**Background and aim:** Nutritional deficiencies are frequent in coeliac disease (CeD), mostly because of the nutritional deficits in gluten-free foods and because of wrong behaviors. We aimed to investigate the level of nutritional knowledge in a cohort of CeD patients in comparison with patients with inflammatory bowel disease (IBD) and healthy subjects. **Materials and methods:** We consecutively recruited CeD patients and matched-sex and -age IBD patients between April and December 2019 at the University Hospital of Padua outpatient clinic. Healthy subjects were also recruited from family and friends of the hospital staff. The CeD patients were asymptomatic on a gluten-free diet, whereas the IBD patients were in remission. All of the subjects completed the Moynihan validated questionnaire to measure their nutritional knowledge. **Results:** We included 96 CeD patients, 96 IBD patients, and 65 healthy controls. We found that CeD patients were less aware of nutritional recommendations compared with healthy subjects (HS), and were less able to identify nutrient sources compared with IBD patients and to choose healthy food compared with both groups. The Moynihan questionnaire mean total score was not significantly different between CeD and IBD groups (mean 22.5 ± 2.3 for CeD, 22.0 ± 2.2 for IBD), while it was statistically significantly worse in CeD compared with healthy subjects (mean 21.2 ± 2.3 for HS, *p* = 0.001). **Conclusions:** CeD patients tend to focus their diet on gluten avoidance, while IBD patients tend to follow a healthier diet, probably because they believe that diet plays a major role in regulating inflammation and, therefore, their symptoms. A dietitian consultation at CeD diagnosis is recommended.

## 1. Introduction

Coeliac disease (CeD) is an autoimmune multi-organ disease affecting the small bowel in genetically predisposed persons, precipitated by the ingestion of gluten [1,2]. The mainstay treatment is based on the exclusion of gluten from the diet, so CeD patients should be educated to avoid any foods derived from wheat, barley, or rye [1]. The dietary changes imposed by the disease may contribute to a low quality of life and sleep disturbances reported during follow up by CeD patients, similarly to what happens in other gastrointestinal disorders [3,4,5,6,7]. According to the CeD guidelines [1,8], patients should be referred to a dietitian to discuss dietary management, so as to avoid gluten contamination and nutritional deficiencies. Gluten-free diet (GFD) has been in fact associated with possible diet imbalances [9], which could contribute to the higher risk of metabolic syndrome and hepatic steatosis [10,11] described in recent literature in CeD patients at follow-up. Dietary imbalance on a GFD might be related to the general nutritional knowledge of CeD patients, which, based on our knowledge, has never been investigated.

Therefore, we aimed to investigate the level of nutritional knowledge in a cohort of adult CeD patients compared with sex- and age-matched patients with inflammatory bowel disease (IBD) and healthy subjects. The IBD patients were selected as a comparison group [12] because of their perception of diet as a key factor in their disease management resulting in many of them following a strict diet in order to reduce abdominal symptoms and prolong remission state, despite no studies having demonstrated that particular foods cause increased inflammation and risk of relapse [13,14].

## 2. Materials and Methods

We consecutively and prospectively recruited all adult CeD patients who visited the CeD Unit of the University Hospital of Padua from April to December 2019. We also included age- and sex-matched IBD patients who visited the IBD Unit in the same period of time, as well as a group of healthy subjects (HS) from the family and friends of the hospital staff. For CeD patients, inclusion criteria were age ≥18 years; a CeD diagnosis from at least one year ago, based on serology and histological evaluation; proper compliance to treatment with GFD; and negative CeD serology at follow-up. For IBD patients, inclusion criteria were age ≥18 years and a confirmed diagnosis of ulcerative colitis (UC) or Crohn’s disease (CD) based on clinical, endoscopic, and histological examinations, according to international criteria, from at least the last six months [15]. All IBD patients needed to be in a remission state, based on a partial Mayo Score (pMayo) <2 for UC patients and a Harvey-Bradshaw index (HBI) <5 for CD patients at the moment of the evaluation. We opted to include only remission patients in order to limit the influence of disease activity on the answers given, and to homogenize this control population with that of CeD (i.e., all in remission and in GFD). For all of the groups, exclusion criteria were the presence of symptoms or signs of disease activity at the outpatient visit, inability to complete a questionnaire, significant psychiatric diagnoses (including dementia), an ongoing hypocaloric diet based on a dietitian suggestion, a medical education background, history of alcoholism, or refusal to sign the informed consent form. All participants were informed about the nature, duration, and purpose of the study. Demographics and clinical information were taken from the outpatient medical records and/or in collaboration with the patient. All of the subjects were asked to complete the Moynihan validated questionnaire, translated and adapted to the Italian language and eating habits, [16,17] in order to measure nutritional knowledge in the following four main domains: dietary recommendations (four questions), nutrient sources (two questions), ability to select healthy meal options (four questions), and associations between diet and diseases (one question). The questionnaire was composed of single- and multiple-choice questions. We reported the percentage of correct answers to the single-choice questions and the mean of the correct options chosen for the multiple-choice questions. A higher total and sub score correspond to a lower nutritional knowledge. The ethical approval codes are 4680/AO/19 and 4197/AO/17.

### Statistical Analysis

All of the analyses were performed using STATA 11 software (Stata Corp., College Station, TX, USA). Categorical and continuous variables were expressed as frequency and mean ± standard deviation (SD), respectively. Chi-square and Student’s *t*-test were used to compare the categorical and continuous analyses, respectively.

## 3. Results

We included 96 CeD patients, 96 IBD patients, and 65 healthy controls (Table 1). None of the patients selected refused to participate in the study. Patients with CeD were similar to the other two groups in terms of age, gender, educational status, and body mass index (BMI). Moreover, the CeD patients were on a GFD from a mean of 6.8 (SD 6.4) years, whereas patients with IBD and HS were not following any strict or specific diet.

### 3.1. Nutritional Knowledge in CeD Compared to IBD

As illustrated in Table 2, the overall dietary recommendation knowledge tended to be worse in CeD patients compared with IBD patients (*p* = 0.09). We observed a higher unawareness of the correct amount of foods people should eat to achieve an ideal daily balance in CeD compared with IBD patients (question 1, *p* = 0.005). In particular, we observed, through the first question, an unawareness of high fruit and vegetable intake benefits in CeD compared with IBD patients (correct response: 71.9% CeD and 87.5% IBD; *p* = 0.007). More IBD patients than CeD were aware of the recommendations given by the experts on which foods to consume more or less of (question 4, *p* = 0.01). Regarding nutrient sources, fewer IBD patients were able to identify the dietary source of oily fish compared with CeD patients (correct response: 4.2% IBD, 17.7% CeD; question 5, *p* = 0.003); while more CeD patients incorrectly identified the foods containing fiber (question 6, *p* = 0.03). In particular, more CeD patients incorrectly identified fish and chicken as foods high in fiber, compared with IBD patients (correct response: 67.7% CeD, 83.3% IBD, *p* = 0.01; 72.9% CeD, 85.4% IBD, *p* = 0.03; respectively). Finally, more IBD patients identified the healthiest choice from different types of pasta with sauce (correct response: 52.1% CeD, 68.7% IBD, question 9, *p* = 0.02). No significant difference was found in the knowledge of potential associations between diet and different diseases between the groups. The Moynihan questionnaire mean total score was not significantly different between the two groups (mean 22.5 ± 2.3 for CeD and 22.01 ± 2.2 for IBD).

### 3.2. Nutritional Knowledge in CeD Compared to Controls

As shown in Table 2, the dietary recommendation knowledge was statistically worse in CeD patients compared with the controls (mean ± SD: 6.3 ± 0.6 for CeD and 5.9 ± 0.7 for controls; *p* = 0.008), principally due to the higher unawareness of the correct amount of food people should eat in order to achieve an ideal daily balance (question 1, *p* = 0.003). Regarding the nutrient sources, no significant difference was found between the groups. More controls, compared with CeD patients, identified the healthiest meal options among different sandwiches (question 7, *p* = 0.02) and types of pasta with sauce proposed (question 9, *p* = 0.003). A significant difference was found in the knowledge of associations between diet and diseases among the groups, with HS being more able to identify these associations (mean ± SD: 7.68 ± 1.3 for CeD and 7.15 ± 1.2 for controls; *p* = 0.03). Finally, the Moynihan questionnaire mean total score was found to be significantly different between the groups (mean 22.5 ± 2.3 for CeD and 21.2 ± 2.3 for HS; *p* = 0.001).

## 4. Discussion

This cross-sectional study aims to evaluate the nutritional knowledge of CeD patients on a GFD, and to compare it with that of patients with a different chronic intestinal inflammatory disease and a group of healthy subjects. We found that CeD patients are less aware of nutritional recommendations compared with healthy subjects, and are less able to identify nutrients sources compared with IBD patients, as well as to choose healthy foods compared with both groups. 

CeD treatment is focused on gluten-containing food avoidance, and less importance has been given to the nutritional quality of this diet [18]. Dietary insufficiencies are frequent in CeD, and may relate to wrong behaviour in addition to inherent deficiencies in the GFD [19]. A study in children showed that the intakes of simple sugars, fats, and protein exceeded the national recommendations for health [20]. The dietary imbalance could contribute to the higher prevalence of the increase in waist circumference, hypertension, reduction of HDL cholesterol, hyperglycaemia, hypercholesterolemia, BMI > 25, and hepatic steatosis after GFD compared with baseline at CeD diagnosis, recently reported [11].

Therefore, at diagnosis, doctors should focus not only on dietary avoidance, but also on a balanced diet. Our results are in support of a recent review [9] on nutritional deficiencies in CeD, which concludes that the early education of patients by a dietitian with expert knowledge in CeD is needed in order to address the achievement of an adequate nutrient intake. Based on our results, the dietitian consultation should also aim to increase patients’ nutritional knowledge. An Italian study reported a higher risk of pathological eating behaviour in CeD patients at diagnosis [21]. In particular, the authors found that the consumption of pasta and bread was approximately 30% higher in CeD at diagnosis than in healthy subjects, with the consumption of fruits and vegetables slightly lower in CeD than in the healthy controls. Moreover, the authors found bulimic behaviour in CeD men. In this context, a dietitian consultation becomes even more relevant.

Regarding IBD, there are multiple diets that have been implicated in IBD treatment (exclusion enteral nutrition, specific carbohydrate diet, low FODMAP, Mediterranean, etc.), however evidence on them is very limited [14]. IBD patients view diet as a crucial component in the management of their IBD, and more than half of them report not receiving any information about it [22,23]. Despite the lack of information, our study showed that IBD patients tend to have a better dietary knowledge compared with CeD patients.

The main strength of our study is that, for the first time, we evaluated nutritional knowledge using a validated questionnaire in a large cohort of CeD patients, comparing the results to those obtained in a cohort of patients with another immune-mediated disease involving the gastrointestinal tract, and a group of healthy subjects. We matched the patients for sex and age so as to avoid any possible influence of these factors, and we did not observe any difference in terms of education that might have impacted subjects’ knowledge. A possible limitation of our study is the lack of evaluation of blood nutrient levels, which would give us a real perception of the nutritional deficiencies correlated to nutritional knowledge.

In conclusion, we found that CeD patients tend to focus on gluten avoidance rather than trying to follow a balanced healthy diet in order to prevent other potential diseases, while IBD patients tend to follow a healthier diet, probably because they believe that diet plays a major role in regulating inflammation and, therefore, their symptoms. A dietitian consultation at CeD diagnosis is recommended.

## Figures and Tables

**Table 1:** Demographic and clinical characteristics of the patients and controls at baseline.

	CeD Patients	IBD Patients	Healthy Subjects	*p* CeD vs. IBD	*p* CeD vs. HS
N	96	96	65		
Mean age, *n* ± SD	39.56 (13.55)	43.16 (12.53)	40.04 (11.9)	0.2	0.4
Female gender, *n* (%)	82 (85.4)	72 (75)	48 (73.8)	0.07	0.07
Educational status, *n* (%)					
Middle School	8 (8.3)	6 (6.2)	10 (15.4)	0.85	0.37
High School	56 (58.3)	58 (60.4)	35 (53.8)		
University	32 (33.4)	32 (33.4)	20 (30.8)		
BMI, *n* (%)					
<16.5	1 (1.)	0	0	0.07	0.2
16.5–18.49	5 (5.2)	12 (13.8)	3 (4.6)		
18.5–24.49	71 (74)	49 (56.3)	40 (61.6)		
25–29.99	13 (13.6)	21 (24.1)	19 (29.2)		
30–34.99	5 (5.2)	5 (5.8)	3 (4.6)		
≥35	1 (1)	0	0		

**Table 2:** Knowledge assessed: Coeliac disease (CeD) vs. irritable bowel disease (IBD), and CeD vs. healthy subjects.

	Score Range	CeD *n* = 96	IBD *n* = 96	HS *n* = 65	*p* CeD vs. IBD	*p* CeD vs. HS
**Dietary Recommendation—mean (SD)**	**4–8**	**6.30 (0.59)**	**6.1 (0.77)**	**5.9 (0.74)**	0.09	0.008
1. Awareness of the recommended proportions of foods to achieve the ideal daily balance—mean (SD)		1.57 (0.23)	1.48 (0.21)	1.46 (0.19)	0.005	0.003
2. Awareness of how many servings of fruit and vegetables experts suggest to eat every day—% of correct answers		15.6	19.8	27.7	0.28	0.06
3. Awareness of types of fat experts recommend to cut down—% of correct answers		51.0	55.2	63.1	0.56	0.13
4. Awareness of which foods experts recommend to eat less, same or more—mean (SD)		1.39 (0.11)	1.35 (0.14)	1.40 (0.13)	0.01	0.75
**Nutrient Source—mean (SD)**	**2–4**	**3.10 (0.45)**	**3.17 (0.29)**	**3.03 (0.52)**	0.4	0.85
5. Knowledge of the main dietary sources of oily fish—% of correct answers		17.7	4.2	21.5	0.003	0.54
6. Knowledge of foods containing high and low amounts of dietary fibre—mean (SD)		1.28 (0.21)	1.22 (0.19)	1.24 (0.23)	0.03	0.33
**Healthiest Meal Option—mean (SD)**	**4–8**	**5.42 (1.03)**	**5.31 (1.04)**	**5.01 (0.9)**	1	0.04
7. Healthiest sandwich option—% of correct answers		51.0	58.3	69.2	0.31	0.02
8. Best choice for high fibre and low-fat meal—% of correct answers		64.6	53.1	66.1	0.10	0.8
9. Healthiest pasta with sauce option—% of correct answers		52.1	68.7	75.4	0.02	0.003
10. Best low-fat grilled meat option—% of correct answers		90.6	88.5	87.7	0.63	0.5
**Knowledge about the association between diet and disease—mean (SD)**	**5–10**	**7.68 (1.30)**	**7.44 (1.28)**	**7.15 (1.2)**	0.64	0.03
11. Association between disease and low fibre intake—% of correct answers		19.8	19.8	16.9	1	0.64
11a. Association between disease and fruit and vegetables intake—% of correct answers		8.3	12.5	23.3	0.34	0.009
11b. Association between disease and fat intake—% of correct answers		68.8	76.04	84.6	0.25	0.02
11c. Association between disease and sugar intake—% of correct answers		77.1	78.1	92.3	0.86	0.01
11d. Association between disease and salt intake—% of correct answers		58.3	68.7	67.7	0.38	0.23
**Total score—mean (SD)**	**15–30**	**22.5 (2.27)**	**22.01 (2.20)**	**21.16 (2.3)**	0.41	0.001

Note: The score for each domain is given by the sum of the scores of all of the questions within the domain. Correct answers have a score of 1, while the wrong answers have score of 2. For multiple-choice question number 1, which has 5 options, the score is 0.2 for each correct option selected, and 0.4 for each wrong option selected. For multiple choice questions number 4 and 6, which have 10 options, the score is 0.1 for each correct option selected and 0.2 for each wrong option selected. Therefore, a higher score corresponds to a lower knowledge. The questionnaire is available online in English [16] and Italian [17] language versions.
